# An effective and chemotherapy-free strategy of all-trans retinoic acid and arsenic trioxide for acute promyelocytic leukemia in all risk groups (APL15 trial)

**DOI:** 10.1038/s41408-022-00753-y

**Published:** 2022-11-21

**Authors:** Huai-Yu Wang, Sha Gong, Guo-Hui Li, Ya-Zhou Yao, Yin-Suo Zheng, Xiao-Hong Lu, Su-Hua Wei, Wei-Wei Qin, Hai-Bo Liu, Meng-Chang Wang, Jie-Ying Xi, Li-Mei Chen, Mei Zhang, Xin-Xin Zhang, Hui-Yun Zhang, Cheng-Sheng Zhang, David N. Wald, Hong-Hu Zhu, Li Liu, Peng-Cheng He

**Affiliations:** 1 Department of Hematology, The First Affiliated Hospital of Xi’an Jiaotong University, Xi’an, Shaanxi Province China; 2 Department of Hematology, Tangdu Hospital, Air Force Medical University, Xi’an, Shaanxi Province China; 3 Department of Hematology, Baoji Central Hospital, Baoji, Shaanxi Province China; 4 Department of Rheumatology, The First Affiliated Hospital of Xi’an Jiaotong University, Xi’an, Shaanxi Province China; 5 Department of Oncology, Qinghai Provincial People’s Hospital, Xining, Qinghai Province China; 6 Precision Medicine Center, The First Affiliated Hospital of Xi’an Jiaotong University, Xi’an, Shaanxi Province China; 7 Department of Pathology, Case Western Reserve University, Cleveland, OH USA; 8 Department of Hematology, The First Affiliated Hospital, Zhejiang University School of Medicine, Hangzhou, Zhejiang China

**Keywords:** Acute myeloid leukaemia, Randomized controlled trials

## Abstract

The combination of all-trans retinoic acid (ATRA) and arsenic trioxide (ATO) has been demonstrated to have comparable effectiveness or better to ATRA and chemotherapy (CHT) in non-high-risk acute promyelocytic leukemia (APL). However, the efficacy of ATRA-ATO compared to ATRA-ATO plus CHT in high-risk APL remains unknown. Here we performed a randomized multi-center non-inferiority phase III study to compare the efficacy of ATRA-ATO and ATRA-ATO plus CHT in newly diagnosed all-risk APL to address this question. Patients were assigned to receive ATRA-ATO for induction, consolidation, and maintenance or ATRA-ATO plus CHT for induction followed by three cycles of consolidation therapy, and maintenance therapy with ATRA-ATO. In the non-CHT group, hydroxyurea was used to control leukocytosis. A total of 128 patients were treated. The complete remission rate was 97% in both groups. The 2-year disease-free, event-free survival rates in the non-CHT group and CHT group in all-risk patients were 98% *vs* 97%, and 95% *vs* 92%, respectively (*P* = 0.62 and *P* = 0.39, respectively). And they were 94% *vs* 87%, and 85% *vs* 78% in the high-risk patients (*P* = 0.52 and *P* = 0.44, respectively). This study demonstrated that ATRA-ATO had the same efficacy as the ATRA-ATO plus CHT in the treatment of patients with all-risk APL.

## Introduction

Acute promyelocytic leukemia (APL) is a subtype of acute myeloid leukemia (AML) characterized by the t(15;17)(q22;q12) translocation, resulting in the fusion of the promyelocytic leukemia (PML) gene and retinoic acid receptor alpha (RARα) gene (*PML-RARα*) [1]. Historically, APL was the leukemia subtype with the highest mortality due to serious coagulopathies in these patients [2]. The use of daunorubicin markedly improved the complete remission rate of APL patients from ~13 to 55% [3]. In contrast, the combination of all-trans retinoic acid (ATRA) and chemotherapy (CHT) resulted in complete remission rate of up to 90% [4]. This remarkable improvement has led to the combination of ATRA and CHT becoming the standard treatment for APL for over a decade [5, 6]. However, anthracycline-based CHT has several disadvantages, including severe bone marrow suppression, subsequent fatal infections and therapy-related neoplasms, which encourage scientists to explore CHT-free or CHT-reducing strategy in order to cure APL [7–9].

In addition to ATRA, arsenic trioxide (ATO) and gemtuzumab ozogamicin (GO) have been found to be highly effective in the treatment of APL [10–14]. A study from the MD Anderson Cancer Center and the AML17 trial from UK MRC demonstrated that ATRA-ATO plus GO was effective and safe, especially in high-risk APL [7, 15]. However, GO is not yet commercially available in China and many developing countries, and its high price limits its wide application.

Compared with ATRA-CHT, the CHT-free Strategy of ATRA and ATO resulted in greater event-free survival in non-high risk APL in the APL0406 trial [8, 16, 17]. However, this study did not include high-risk patients, who account for roughly 25% of APL patients. Moreover, the control group in this study was ATRA-CHT rather than ATRA-CHT plus ATO, which was demonstrated to be superior to ATRA-CHT in all-risk APL patients in the C9710 and APML4 studies [18–20]. Therefore, we performed a randomized, multi-center non-inferiority clinical trial (APL15) to directly compare the efficacy of ATRA-ATO and ATRA-ATO plus CHT in all newly diagnosed APL, to explore the necessity of chemotherapy, especially for high-risk patients.

## Methods

### Study design and patients

This study is a randomized, prospective, multicenter, phase III non-inferiority trial done at three hospitals in China. The initial full trial design was published in 2018 [21]. Briefly, eligible patients were 15 to 80 years old with newly diagnosed, low- or high-risk APL. Initial patient enrollment was solely based on cytomorphologic features according to the French-American-British (FAB) criteria [22]. Genetic diagnosis of APL was confirmed by reverse-transcriptase polymerase chain reaction (RT-PCR) for the presence of the *PML-RARα* fusion transcript [23, 24] or the presence of t(15;17) via standard karyotyping or fluorescence in-situ hybridisation (FISH) on bone marrow aspirates [25]. Additional inclusion criteria were a serum total bilirubin concentration of up to three times the maximum institutional upper limit of normal (ULN) and a serum creatinine concentration of up to 2.5 times the maximum ULN. Exclusion criteria were pregnancy, lactation, concomitant severe psychiatric disorder, significant arrhythmias, and other active malignancies.

This study was approved by the Ethics Committee of The First Affiliated Hospital of Xi’an Jiaotong University in Xi’an, China (Approval no. 2015-012). The patients were enrolled from July 2015 to January 2021. Written informed consent was obtained from all patients before study entry. The trial was registered with the Chinese Clinical Trial Registry (ChiCTR-IPR-15006821) and conducted in accordance with the Declaration of Helsinki.

### Treatment arms

**Fig. 1:**
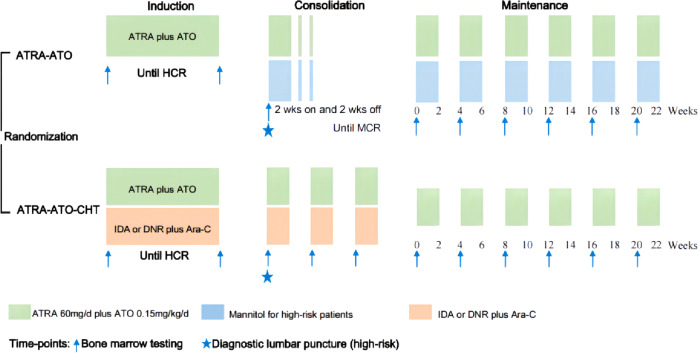
Study design. ATRA all-trans retinoic acid. ATO arsenic trioxide, CHT chemotherapy, HCR hematological complete remission, MCR Molecular complete remission. In the ATRA-ATO group, synchronous administration of mannitol and ATO was used to prevent central nervous system leukemia in high-risk patients. In the ATRA-ATO-CHT group, the chemotherapy regimen was as follows: IDA (idarubicin) at a dose of 8 mg/m^2^/day on days 2, 4, 6 or DNR (daunorubicin) at a dose of 45 mg/m^2^/day on days 2, 4, 6, 8 plus Ara-C(cytosine arabinoside) at a dose of 150 mg/m^2^/day on days 1-7. The bone marrow testing included morphology and molecular evaluation.

The treatment schedules for the ATRA-ATO (non-CHT group) and ATRA-ATO plus CHT (CHT group) regimens have been described previously [21] and are shown in Fig. 1. All patients were assigned to receive ATRA-ATO for induction, consolidation, and maintenance or ATRA-ATO plus CHT induction therapy followed by three cycles of consolidation therapy with ATRA-ATO plus CHT, and maintenance therapy with ATRA-ATO for 6–10 cycles. To streamline the drug regimen, ATRA (Shandong Longfine Pharmaceutical Co., Ltd, China) was given at 40 mg/d (BSA < 1.5 m^2^) or 60 mg/d (BSA ≥ 1.5 m^2^) (20–45 mg/m^2^/d) in divided doses in both groups since previous studies from the Hospital Saint Louis (French) [26] and the Shanghai Institute of Hematology (China) [27] reported that the lower ATRA dose (20 mg/m^2^/d)) had the same therapeutic effects as the conventional dose of 45 mg/m^2^/d. ATO was given at 0.15 mg/kg/d. In previous trials, ATRA was used for 2 weeks every 4 weeks, and ATO was used for 4 weeks every 8 weeks in post-remission treatment [8, 28]. To simplify the treatment strategy, both ATRA and ATO were administered for 2 weeks every 4 weeks in the consolidation and maintenance therapy in this study. In the CHT group, the chemotherapy regimen was as follows: idarubicin (IDA) at a dose of 8 mg/m^2^/day on days 2, 4, 6 or daunorubicin (DNR) at a dose of 45 mg/m^2^/day on days 2, 4, 6, 8 plus cytosine arabinoside (Ara-C) at a dose of 150 mg/m^2^/day on days 1–7. Chemotherapy doses could be reduced according to patient’s performance status.

Diagnostic lumbar puncture was routinely done at the first course in consolidation with high-risk patients, but not in the low-risk patients. High-risk patients in CHT group received central nervous system prophylaxis with intrathecal chemotherapy at least once after HCR. Synchronous administration of mannitol and ATO was used to increase the permeability of the blood brain barrier (BBB) in the consolidation and maintenance, thereby improving the concentration of ATO in the cerebrospinal fluid (CSF) and preventing central nervous system leukemia (CNSL) in the ATRA-ATO treated high-risk patients. In induction therapy, a bone marrow examination to assess the response was performed when the absolute neutrophil count was more than 1.0 × 10^9^/L and the platelet count was more than 100 × 10^9^/L. Once in complete remission (CR), the disease monitoring and management of minimal residual disease were performed as previously reported [21].

### Supportive measures and management of complications

All patients received blood product transfusions to maintain platelet counts above 30 × 10^9^/L and serum fibrinogen levels above 1.5 g/L. Hydroxyurea was the only cytotoxic agent allowed to control hyperleukocytosis in the non-CHT group. Patients received hydroxyurea at 1–4 g/d according to the initial study design, but after 2018, hydroxyurea was given at 4 g/d when WBC count >10 × 10^9^/L [21]. The maximum hydroxyurea dose could be up to 0.1 g/kg/d in high-risk patients if the WBC count consistently increased.

Prophylaxis for differentiation syndrome (DS) was not recommended, but dexamethasone was administered at a dose of 10 mg every 12 h intravenously at the earliest manifestations of suspected DS until the disappearance of signs and symptoms. Temporary discontinuation and dose adjustments were recommended to manage drug-related hematologic and nonhematologic toxicities.

### Definitions and study end points

Patients were risk-stratified based on the WBC count at diagnosis using a modified classification into a non-high-risk group (WBC < 10 × 10^9^/L) and a high-risk group (WBC ≥ 10 × 10^9^/L) [6]. Leukocytosis was defined as a WBC count of more than 10 × 10^9^/L [16].

The primary end point was event-free survival (EFS) and disease-free survival (DFS) at 2 years. EFS was defined as the time from diagnosis to first event, including death during induction therapy, failure to achieve remission, death during remission, relapse at any site, or the development of second malignant neoplasm. DFS was defined as the time from hematological complete remission (HCR) to either hematological or molecular relapse or death from APL. The secondary end points included CR, and overall survival (OS) at 2 years. Hematological complete remission and relapse were defined according to criteria described by the National Cancer Institute (NCI) workshop [29]. Molecular complete remission (MCR) was defined as the absence of detectable nuclear transcripts, and molecular relapse was defined as the reversion to positivity in two consecutive bone marrow samples performed at least 2 weeks apart [23]. OS was defined as the time from diagnosis to death. Treatment-related toxicities were graded according to the NCI Common Terminology Criteria for Adverse Events, version 4.0 (CTCAE4). The monitoring of hematological and nonhematological adverse events details was performed as previously reported [21].

### Statistical analysis

This trial was designed to evaluate the non-inferiority of DFS in the non-CHT group compared with the CHT group for 2 years following the initiation of induction therapy. We chose 10% as the non-inferiority margin, a type I error probability of 5%, and a power of 80%. All patients enrolled in the study were analyzed following an intent-to-treat principle. Patient characteristics were summarized by crosstab. Chi-square test or Fisher’s exact test were used for comparison of categorical variables, and *t* test or Mann–Whitney *U* test for continuous variables. The survival distributions were estimated by the Kaplan–Meier method and compared with the use of log-rank test between the two groups. All statistical tests were two-sided with a significance level of 0.05, except the non-inferiority hypothesis. The data were analyzed using SPSS software (version 23.0) and R software (version 4.0.4).

## Results

### Patient characteristics

**Fig. 2:**
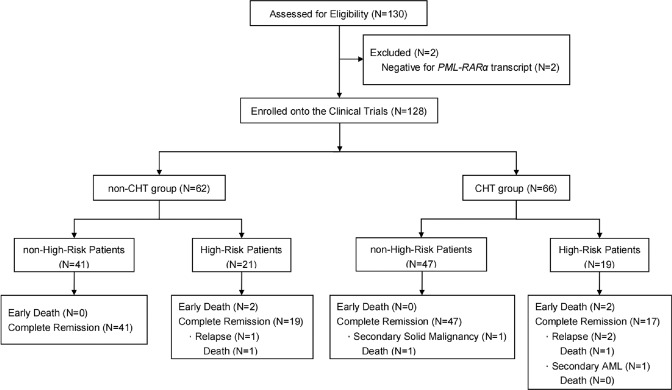
Trial profile. CHT chemotherapy.

A total of 130 patients with newly diagnosed APL were enrolled from July 2015 to January 2021 and assigned to either the non-CHT group (*n* = 63) or CHT group (*n* = 67) (Fig. 1). Two patients (one in the non-CHT group and one in the CHT group) were excluded that tested negative for the *PML-RARα* fusion transcript. The remaining 128 patients were included in the intent-to-treat analysis, of which 40 were high-risk patients (21 in the non-CHT group and 19 in the CHT group). The median age of the participants was 40.5 years (age range: 15–69) and 13 were older than 60 years. The median WBC count at presentation was 2.93 × 10^9^/L (0.31–163.42 × 10^9^/L). The major clinical and biological characteristics of these 128 patients are shown in Table 1. There were no significant differences in the baseline characteristics between the two cohorts. The trial profile was provided in Fig. 2. The present analysis was performed in February 2022 with a median follow-up time of 45.22 months (0.1–78.27 months).

**Table 1:** Patient characteristics at diagnosis.

Characteristic	non-CHT group (*n* = 62)	CHT group (*n* = 66)	*P*
Median age, years (range)	41 (15–69)	40.5 (15–65)	0.82
Sex, No. (%)			0.87
Male	32 (51.6)	35 (53.0)	
Female	30 (48.4)	31 (47.0)	
Median WBC, ×10^9^/L (range)	2.93 (0.34–163.42)	3.03 (0.31–150.40)	0.60
Median PLT, ×10^9^/L (range)	28 (3–113)	24 (2–249)	0.31
Sanz risk, No. (%)			0.54
Low and intermediate	41 (66.1)	47 (71.2)	
High	21 (33.9)	19 (28.8)	
*PML/RARα* isoform, No. (%)			0.41
BCR1–2	36 (58.1)	43 (65.2)	
BCR3	26 (41.9)	23 (34.8)	
*FLT3-*ITD, No. (%)			0.69
Mutated	17 (27.4)	22 (33.3)	
Unmutated	35 (56.5)	36 (54.5)	
Missing	10 (16.1)	8 (12.1)	
Low fibrinogen, No. (%)	35 (56.5)	28 (42.4)	0.11
Intracranial hemorrhage, No. (%)	3 (4.8)	2 (3.0)	0.67

*CHT* chemotherapy, *PML/RARα* promyelocytic leukemia/retinoic acid receptor alpha, BCR breakpoint cluster regions, *FLT3*-ITD Fms-like tyrosine kinase 3-internal tandem duplication.

### Induction therapy

A total of 62 patients in the non-CHT group and 66 patients in the CHT group were evaluable for induction response. HCR was achieved in 60 of 62 patients in the non-CHT group (97%) and 64 of 66 patients in the CHT group (97%) (*P* = 1.00). Two patients in the non-CHT group died on day 3 and day 4, respectively, due to intracranial hemorrhage during the induction therapy. Two patients died in the CHT group: one died of bronchopneumonia on day 11 and another died of intracranial hemorrhage on day 17. All four patients that died were in the high-risk group. HCR was 90% in the non-CHT group and 89% in the CHT group in high-risk patients (*P* = 1.00). Both groups in the non-high-risk patients experienced 100% HCR. Of the 124 patients, the median time to HCR was 32.5 days (14–54 days) in the non-CHT group and 34 days (21–51 days) in the CHT group (*P* = 0.64).

115 patients (56 in the non-CHT group and 59 in the CHT group) received ATRA 60 mg/d and 13 patients (6 in the non-CHT group and 7 in the CHT group) received 40 mg/d. Ten patients in the non-CHT group (16%) and 11 patients in the CHT group (17%) developed mild DS (*P* = 0.87), however none of the patients exhibited severe DS. There were no significant differences in the incidence rate and degree of DS among patients with ATRA at 40 mg/d or 60 mg/d. Leukocytosis developed during induction therapy in 32 of 41 non-high-risk patients in the non-CHT group (78%) and 42 of 47 non-high-risk patients in the CHT group (89%) (*P* = 0.15). All cases of DS in the non-CHT group were successfully managed with hydroxyurea using the recommended doses in the protocol. For non-high-risk patients, the median WBC count peak was 20.39 × 10^9^/L (1.49–96.10 × 10^9^/L) in the non-CHT group and 24.47 × 10^9^/L (4.48–96.15 × 10^9^/L) in the CHT group (*P* = 0.45). The median time to develop leukocytosis was 4.5 days (2–15 days) and it took a median time of 9 days (2–16 days) to control it. To restore WBC counts within normal range with hydroxyurea. A mean dose of 33.71 mg (9–52 mg) hydroxyurea was needed to control the WBC count. For high-risk patients, the mean dose of hydroxyurea was 36.03 g (19.5–59 g), and the median time it took to control WBC count was 13 days (5–16 days). Previous DIC lab values and supportive care did not change during the hydroxyurea treatment. No obvious side effects were observed from using hydroxyurea during its administrations. There were no significant differences in length of hospital stay or IV antibiotics requirements between the non-CHT and CHT groups.

### Consolidation therapy

A total of 118 of the 124 patients in HCR proceeded to consolidation therapy. In the non-CHT group, one high-risk patient did not receive any post-remission therapy and disease monitoring. Two patients (one with high-risk APL and one with non-high-risk APL) did not receive the assigned treatment but received ATRA and Realgar-Indigo naturalis formula (RIF) due to the patients’ choice. In the CHT group, three patients only received ATRA-ATO in consolidation therapy because of patients’ choice. However, all patients mentioned above were evaluated for the primary end point.

59 patients in the non-CHT group and 62 patients in the CHT group were evaluated for a molecular response and all achieved MCR. The median time to MCR was 55 days (35–85 days) in the non-CHT group and 59 days (41–95 days) in the CHT group (*P* = 0.20).

### Maintenance therapy

A total of 118 patients completed the consolidation therapy described above, 115 of which proceeded to maintenance therapy. One non-high-risk patient in the non-CHT group did not receive maintenance therapy for unknown reasons. In the CHT group, one high-risk patient did not receive maintenance therapy for financial reasons and one non-high-risk patient violated the treatment protocol.

### Disease-free survival (DFS)

A total of 124 patients (60 and 64 in the non-CHT and CHT groups, respectively) were assessed for DFS according to the intent-to-treat analysis. Two-year DFS was 98% in the non-CHT group and 97% in the CHT group (*P* = 0.62). The percentage difference in DFS between the two groups was 1.4% (95% CI: 3.8–6.8). The lower limit of the 95% CI for the percentage difference in DFS was greater than the −10% non-inferiority margin, confirming non-inferiority. For high-risk patients, the 2-year DFS was 94 and 87% in the non-CHT group and CHT group, respectively (*P* = 0.52). For non-high-risk patients, the 2-year DFS in both groups was 100%. Overall, three high-risk patients (one in the non-CHT group and two in the CHT group) experienced relapse during follow-up. Of note, the patient in the non-CHT group who did not proceed to post-remission therapy, bone marrow relapsed at 17.4 months from diagnosis. The patient discontinued the re-induction therapy on day 4 due to financial reasons, then died one month later. In the CHT group, the patient who did not complete maintenance therapy had CNSL at 11.2 months. To treat the CNSL, systemic salvage therapy with ATRA/ATO plus chemotherapy and intrathecal chemotherapy plus whole brain radiotherapy were given. However, the patient relapsed again at 17.9 months and died due to disease progression. Another patient experienced hematological relapse at 24.1 months and remained alive after salvage therapy. In the per-protocol population, the 2-year DFS was 100% in the non-CHT group (56 of 56 patients) versus 98% in the CHT group (58 of 59) (*P* = 0.34) in the all-risk patients. The percentage difference in the DFS between the two groups was 2% (95% CI: 1.6–5.0), also confirming non-inferiority.

### Overall survival (OS) and event-free survival (EFS)

**Fig. 3:**
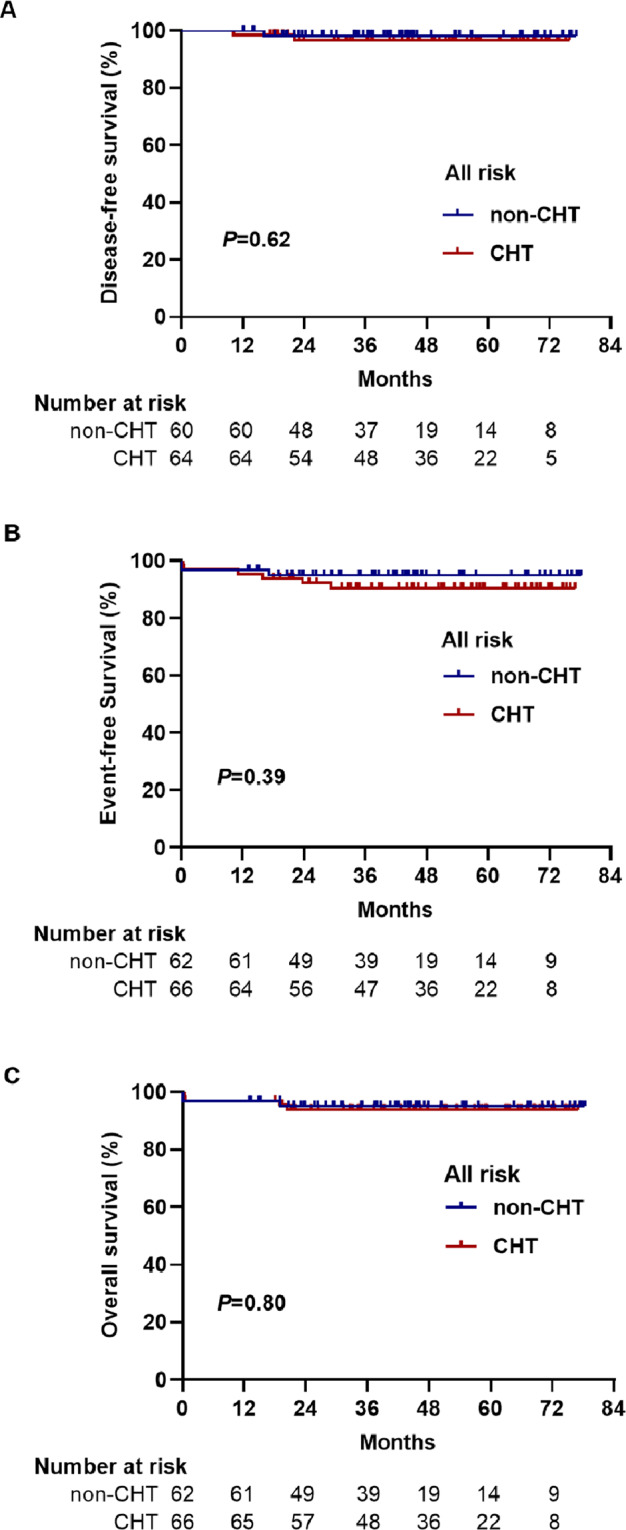
Survival outcomes for all APL patients. **A** Disease-free survival. **B** Event-free survival. **C** Overall survival.

**Fig. 4:**
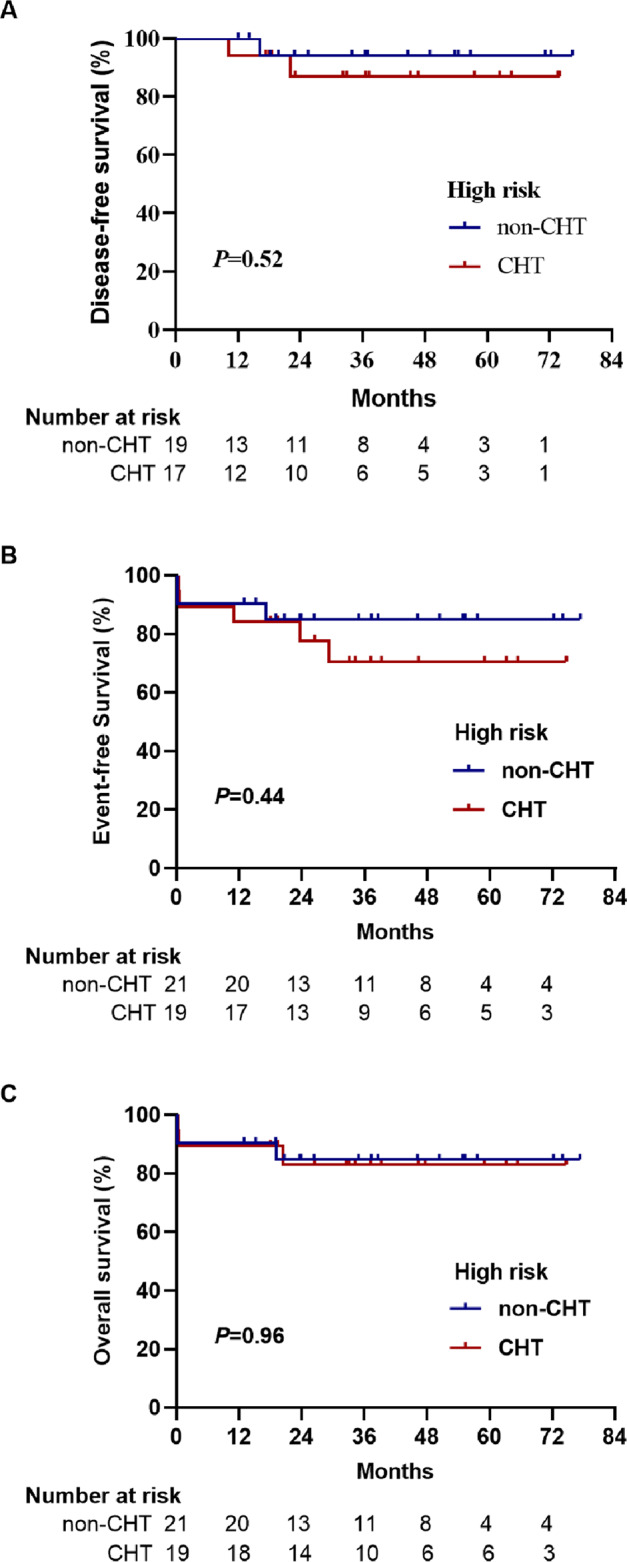
Survival outcomes for high-risk APL patients. **A** Disease-free survival. **B** Event-free survival. **C** Overall survival.

In the intent-to-treat analysis, the 2-year OS was 95% in the non-CHT group and 94% in the CHT group (*P* = 0.80). The 2-year EFS was 95% in the non-CHT group and 92% in the CHT group (*P* = 0.39). The EFS and OS at 2 years for high-risk patients in the non-CHT versus CHT arms were 85% versus 78% (*P* = 0.44) and 85% versus 83% (*P* = 0.96), respectively. Two patients in the CHT group but none in the non-CHT group developed a therapy-related malignancy. One high-risk patient was diagnosed with therapy-related acute myeloid leukemia at 29.7 months and remained alive after intensive chemotherapy. One non-high-risk patient was diagnosed with small cell lung cancer at 16.1 months and died at 19.2 months. In the per-protocol analysis, the 2-year EFS was 97% in the non-CHT group (56 of 58 patients) versus 93% in the CHT group (56 of 61) (*P* = 0.32) in all-risk patients. For high-risk patients, the 2-year EFS was 89% in the non-CHT group (17 of 19 patients) and it was 81% in the CHT group (13 of 17 patients) (*P* = 0.39). The 2-year OS was 97% in the non-CHT group (56 of 58 patients) versus 95% in the CHT group (58 of 61) (*P* = 0.72) in all-risk patients. For high-risk patients, the OS was 89% in the non-CHT group (17 of 19 patients), and it was 88% in the CHT group (15 of 17) (*P* = 0.95). All outcome estimates calculated at 2 years are listed in Table 2, whereas outcome curves are shown in Figs. 3 and 4.

**Table 2:** Clinical outcomes.

	All-risk			Low-risk (*n* = 88)			High-risk (*n* = 40)		
	non-CHT (*n* = 62)	CHT (*n* = 66)	*P*	non-CHT (*n* = 41)	CHT (n = 47)	*P*	non-CHT (*n* = 21)	CHT (*n* = 19)	*P*
*ITT analysis*									
CR	60 (97%)	64 (97%)	1.00	41 (100%)	47 (100%)	–	19 (90%)	17 (89%)	1.00
2-year EFS	59 (95%)	60 (92%)	0.39	41 (100%)	46 (98%)	0.36	18 (85%)	14 (78%)	0.44
2-year DFS	59/60 (98%)	62/64 (97%)	0.62	41 (100%)	47 (100%)	–	18/19 (94%)	15/17 (87%)	0.52
2-year OS	59 (95%)	62 (94%)	0.80	41 (100%)	46 (98%)	0.36	18 (85%)	16 (83%)	0.96
*PP analysis*									
2-year EFS	56/58 (97%)	56/61 (93%)	0.32	39/39 (100%)	43/44 (98%)	0.36	17/19 (89%)	13/17 (81%)	0.39
2-year DFS	56/56 (100%)	58/59 (98%)	0.34	39/39 (100%)	44/44 (100%)	–	17/17 (100%)	14/15 (93%)	0.30
2-year OS	56/58 (97%)	58/61 (95%)	0.72	39/39 (100%)	43/44 (98%)	0.37	17/19 (89%)	15/17 (88%)	0.95
Hospital stay during induction, median days (range)	28 (14–41)	29 (15–47)	0.58	28 (14–41)	28 (15–47)	0.87	29 (16–39)	32 (23–36)	0.38
IV antibiotics during induction, median days (range)	17 (3–31)	19 (7–33)	0.57	16.5 (3–27)	17 (7–33)	0.82	17 (5–31)	18 (14–32)	0.07

*CHT* chemotherapy, *CR* complete remission, *EFS* event-free survival, *OS* overall survival, *DFS* disease-free survival.

### Safety and toxicity

Most severe adverse events occurred during induction therapy. Hematologic and non-hematologic toxicities are listed in Table 3. Grade 3–4 neutropenia lasting more than 15 days occurred in 5 patients (8%) in the non-CHT group and 11 patients (17%) in the CHT group (*P* = 0.14). Grade 3–4 thrombocytopenia (lasting more than 15 days) occurred in 20 patients (32%) in the non-CHT group and 30 patients (45%) in the CHT group (*P* = 0.13). Pneumonia and fever occurred in 31 patients (50%) in the non-CHT group and 35 patients (53%) in the CHT group (*P* = 0.87). Five patients had grade 3–4 hepatic toxicities in each group (*P* = 1.00). Grade 3–4 renal toxicity occurred in one patient in the non-CHT group, while grade 3–4 QTc prolongation occurred in one patient in the CHT group. Grade 3–4 cardiac function was reported in 1 (2%) of 62 patients in the non-CHT group versus 8 (12%) of 68 patients in the CHT group (*P* = 0.03). Skin toxicity mainly occurred during consolidation and maintenance therapy. Grade 1–2 skin toxicities occurred in 9 patients in the non-CHT group and 11 (17%) patients in the CHT group (*P* = 0.74). Grade 3–4 skin toxicity did not occur in either group.

**Table 3:** Incidence of all non-hematological and hematological toxic effects.

Characteristics	non-CHT group (*n* = 62)	CHT group (*n* = 66)	*P*
Differentiation syndrome	10 (16%)	11 (17%)	0.87
Neutropenia (grade 3–4 lasting > 15 days)	5 (8%)	11 (17%)	0.14
Thrombocytopenia (grade 3–4 lasting > 15 days)	20 (32%)	30 (45%)	0.13
Pneumonia and fever	31 (50%)	35 (53%)	0.87
Hepatic toxicity (grade 3–4)	5 (8%)	5 (8%)	1.00
Renal toxicity (grade 3–4)	1 (2%)	0	1.00
Cardiac toxicity (grade 3–4)	1 (2%)	8 (12%)	0.03
QTc prolongation (grade 3–4)	0	1 (2%)	1.00
Skin toxicity (grade 1–3)	9 (15%)	11 (17%)	0.74

Skin toxicity occurred during consolidation and maintenance therapy, all of other toxic effects occurred during induction therapy.

*CHT* chemotherapy.

## Discussion

This trial demonstrates that the combination of ATRA and ATO is non-inferior to ATRA-ATO plus CHT not only in non-high-risk APL, but also in high-risk APL patients. The addition of chemotherapy to ATRA-ATO did not significantly improve the durable anti-leukemia efficacy in these patients. Therefore, a chemotherapy-free regimen combining ATRA plus ATO is a feasible approach to cure all-risk APL patients.

The conventional treatment for APL developed over ten years ago combined ATRA and anthracycline-based chemotherapy to induce remission, followed by consolidation chemotherapy and ATRA maintenance [6, 30–32]. Compared with this classical combination strategy, the C9710 study showed that the addition of ATO as an initial consolidation therapy significantly improved EFS and DFS [18]. Later on, APML4 study introduced ATO to ATRA and anthracycline induction, and eliminated chemotherapy from consolidation [19]. The incorporation of ATO into the initial induction and consolidation therapy for all APL patients reduced the relapse rate when compared with historical controls of APML3 study, which was the ATRA-CHT without ATO strategy [20]. The APL2012 trial from China demonstrated that ATRA-ATO in both chemotherapy-replacing and -reducing settings in consolidation is not inferior to ATRA-CHT [13]. In the above study, ATO was also added in the maintenance therapy. The results of C9710, APML4, and APL2012 strongly supported the application of ATO in all APL patients. AML17 trial explored the combination of ATO and ATO without chemotherapy for all-risk APL patients. The results showed that, ATRA and ATO is effective in low-risk and high-risk patients, with a high cure rate and less relapse when compared with ATRA-CHT group [7, 33]. However, this study added GO to high-risk patients and low-risk patient with leukocytosis in the ATRA-ATO arm. In many countries where chemotherapy is much more affordable than GO, the combination of ATRA-ATO plus chemotherapy is the conventional treatment option. Therefore, we performed a controlled trial to assess the efficacy of ATRA-ATO without chemotherapy and GO for all-risk patients. The results showed that the cure rates and survival rates in the ATRA-ATO group do not differ from the ATRA-ATO plus CHT group for all-risk patients. When compared with APML4 and AML17 trial, the ATRA-ATO group in our study produced similar outcomes. This is the first study that reported all APL patients could be curable without chemotherapy or GO.

The frontline treatment was ATRA-ATO with CHT or GO for high-risk patients according to previous studies [7, 15, 19, 34]. In the single-arm study conducted by MD Anderson Cancer Center, induction with ATRA-ATO plus GO followed by ATRA-ATO consolidation therapy had a 5-year EFS rate of 81% and overall survival rate of 86% for high-risk patients. While in the APML4 with the ATRA-ATO plus CHT strategy, the 5-year EFS and OS were 83 and 87%. In our study, the 2-year event-free and overall survival were 85 and 85% in the non-CHT group. These outcomes were not inferior to CHT group in our study, the historical study of ATRA-ATO plus GO treatment or the ATRA-ATO plus CHT strategy. In addition, patients in the CHT group experienced more cardiac toxicities in our study compared with the non-CHT group. Therefore, we propose that chemotherapy can be removed from the induction and consolidation treatment for high-risk patients.

Hydroxyurea has been shown to significantly reduce WBC counts [35]. The APL0406 trial reported that non-high-risk patients with hyperleukocytosis were successfully treated with hydroxyurea [16]. In this trial, we increased the maximum dose to 0.1 g/kg/d, which has been shown to be safe [36]. The administration of hydroxyurea may achieve the same treatment efficacy as chemotherapy or GO in controlling the WBC count in high-risk patients with APL. Compared with anthracyclines-based chemotherapy, the hydroxyurea dose can be adjusted easily according to the WBC count. In terms of safety, no additional hematological or non-hematological toxicity from hydroxyurea administration was observed. Hydroxyurea may also be a good option to control leukocytosis in CHT-free regimens in high-risk settings or in countries where GO is not available or too expensive.

High-risk APL patients have an increased risk of CNS relapse [37]. It has been shown that ATRA and ATO have the limited ability to penetrate the BBB [38]. As such, intrathecal chemotherapy has been an alternative treatment for CNS prophylaxis. However, its use is still controversial due to the requirement for an invasive procedure and insufficient long term follow-up outcome data [37]. Mannitol has been reported improve arsenic’s ability to penetrate the BBB and reach a CSF concentration 99.7% to that of blood without acute arsenic poisoning [38]. In this trial, mannitol was used in high-risk patients to prevent CNS relapse. In the non-CHT group, none of the patients experienced CNS relapse. Although the role of mannitol in preventing CNS APL still needs to be evaluated, it may be a promising noninvasive CNS prophylactic strategy in high-risk APL.

In conclusion, our results showed that ATRA and ATO without CHT or GO was effective and safe for all-risk APL, suggesting that chemotherapy may be unnecessary for high-risk patients. This work may lead to a new treatment strategy for APL patients, particularly for the high-risk patients. However, more research centers and participants are still needed to further verify this conclusion.

## Data Availability

The data are available from the corresponding author on reasonable request.

## References

[1] de The H, Lavau C, Marchio A, Chomienne C, Degos L, Dejean A (1991). The PML-RAR alpha fusion mRNA generated by the t(15;17) translocation in acute promyelocytic leukemia encodes a functionally altered RAR. Cell.

[2] Hillestad LK (1957). Acute promyelocytic leukemia. Acta Med Scandinavica.

[3] Bernard J, Weil M, Boiron M, Jacquillat C, Flandrin G, Gemon MF (1973). Acute promyelocytic leukemia: results of treatment by daunorubicin. Blood.

[4] Huang ME, Ye YC, Chen SR, Chai JR, Lu JX, Zhoa L (1988). Use of all-trans retinoic acid in the treatment of acute promyelocytic leukemia. Blood.

[5] Avvisati G, Lo Coco F, Diverio D, Falda M, Ferrara F, Lazzarino M (1996). AIDA (all-trans retinoic acid + idarubicin) in newly diagnosed acute promyelocytic leukemia: a Gruppo Italiano Malattie Ematologiche Maligne dell’Adulto (GIMEMA) pilot study. Blood.

[6] Sanz MA, Lo Coco F, Martin G, Avvisati G, Rayon C, Barbui T (2000). Definition of relapse risk and role of nonanthracycline drugs for consolidation in patients with acute promyelocytic leukemia: a joint study of the PETHEMA and GIMEMA cooperative groups. Blood.

[7] Burnett AK, Russell NH, Hills RK, Bowen D, Kell J, Knapper S (2015). Arsenic trioxide and all-trans retinoic acid treatment for acute promyelocytic leukaemia in all risk groups (AML17): results of a randomised, controlled, phase 3 trial. Lancet Oncol.

[8] Platzbecker U, Avvisati G, Cicconi L, Thiede C, Paoloni F, Vignetti M (2017). Improved outcomes with retinoic acid and arsenic trioxide compared with retinoic acid and chemotherapy in non-high-risk acute promyelocytic leukemia: final results of the randomized Italian-German APL0406 trial. J Clin Oncol.

[9] Zhu HH, Liu YR, Jia JS, Qin YZ, Zhao XS, Lai YY (2018). Oral arsenic and all-trans retinoic acid for high-risk acute promyelocytic leukemia. Blood.

[10] Mathews V, Chendamarai E, George B, Viswabandya A, Srivastava A (2011). Treatment of acute promyelocytic leukemia with single-agent arsenic trioxide. Mediterr J Hematol Infect Dis.

[11] Shen ZX, Chen GQ, Ni JH, Li XS, Xiong SM, Qiu QY (1997). Use of arsenic trioxide (As2O3) in the treatment of acute promyelocytic leukemia (APL): II. Clinical efficacy and pharmacokinetics in relapsed patients. Blood.

[12] de Thé H, Le Bras M, Lallemand-Breitenbach V (2012). The cell biology of disease: acute promyelocytic leukemia, arsenic, and PML bodies. J Cell Biol.

[13] Chen L, Zhu HM, Li Y, Liu QF, Hu Y, Zhou JF, et al. Arsenic trioxide replacing or reducing chemotherapy in consolidation therapy for acute promyelocytic leukemia (APL2012 trial). Proc Natl Acad Sci USA*.* 2021;118:e2020382118. 10.1073/pnas.2020382118 PMC8017727 33495363

[14] Takeshita A, Shinjo K, Naito K, Matsui H, Sahara N, Shigeno K (2005). Efficacy of gemtuzumab ozogamicin on ATRA- and arsenic-resistant acute promyelocytic leukemia (APL) cells. Leukemia.

[15] Abaza Y, Kantarjian H, Garcia-Manero G, Estey E, Borthakur G, Jabbour E (2017). Long-term outcome of acute promyelocytic leukemia treated with all-trans-retinoic acid, arsenic trioxide, and gemtuzumab. Blood.

[16] Lo-Coco F, Avvisati G, Vignetti M, Thiede C, Orlando SM, Iacobelli S (2013). Retinoic acid and arsenic trioxide for acute promyelocytic leukemia. N. Engl J Med.

[17] Cicconi L, Platzbecker U, Avvisati G, Paoloni F, Thiede C, Vignetti M (2020). Long-term results of all-trans retinoic acid and arsenic trioxide in non-high-risk acute promyelocytic leukemia: update of the APL0406 Italian-German randomized trial. Leukemia.

[18] Powell BL, Moser B, Stock W, Gallagher RE, Willman CL, Stone RM (2010). Arsenic trioxide improves event-free and overall survival for adults with acute promyelocytic leukemia: North American Leukemia Intergroup Study C9710. Blood.

[19] Iland HJ, Collins M, Bradstock K, Supple SG, Catalano A, Hertzberg M (2015). Use of arsenic trioxide in remission induction and consolidation therapy for acute promyelocytic leukaemia in the Australasian Leukaemia and Lymphoma Group (ALLG) APML4 study: a non-randomised phase 2 trial. Lancet Haematol.

[20] Iland H, Bradstock K, Seymour J, Hertzberg M, Grigg A, Taylor K (2012). Results of the APML3 trial incorporating all-trans-retinoic acid and idarubicin in both induction and consolidation as initial therapy for patients with acute promyelocytic leukemia. Haematologica.

[21] Zhang X, Zhang H, Chen L, Wang M, Xi J, Liu X (2018). Arsenic trioxide and all-trans retinoic acid (ATRA) treatment for acute promyelocytic leukemia in all risk groups: study protocol for a randomized controlled trial. Trials.

[22] Bennett JM, Catovsky D, Daniel MT, Flandrin G, Galton DA, Gralnick HR (1976). Proposals for the classification of the acute leukaemias. French-American-British (FAB) co-operative group. Br J Haematol.

[23] Diverio D, Rossi V, Avvisati G, De Santis S, Pistilli A, Pane F (1998). Early detection of relapse by prospective reverse transcriptase-polymerase chain reaction analysis of the PML/RARalpha fusion gene in patients with acute promyelocytic leukemia enrolled in the GIMEMA-AIEOP multicenter “AIDA” trial. GIMEMA-AIEOP Multicenter “AIDA” Trial. Blood.

[24] van Dongen JJ, Macintyre EA, Gabert JA, Delabesse E, Rossi V, Saglio G (1999). Standardized RT-PCR analysis of fusion gene transcripts from chromosome aberrations in acute leukemia for detection of minimal residual disease. Report of the BIOMED-1 Concerted Action: investigation of minimal residual disease in acute leukemia. Leukemia.

[25] Grimwade D, Biondi A, Mozziconacci M-J, Hagemeijer A, Berger R, Neat M (2000). Characterization of acute promyelocytic leukemia cases lacking the classic t(15;17): results of the European Working Party. Blood.

[26] Castaigne S, Lefebvre P, Chomienne C, Suc E, Rigal-Huguet F, Gardin C (1993). Effectiveness and pharmacokinetics of low-dose all-trans retinoic acid (25 mg/m2) in acute promyelocytic leukemia. Blood.

[27] Chen GQ, Shen ZX, Wu F, Han JY, Miao JM, Zhong HJ (1996). Pharmacokinetics and efficacy of low-dose all-trans retinoic acid in the treatment of acute promyelocytic leukemia. Leukemia.

[28] Zhu HH, Wu DP, Du X, Zhang X, Liu L, Ma J (2018). Oral arsenic plus retinoic acid versus intravenous arsenic plus retinoic acid for non-high-risk acute promyelocytic leukaemia: a non-inferiority, randomised phase 3 trial. Lancet Oncol.

[29] Cheson BD, Bennett JM, Kopecky KJ, Büchner T, Willman CL, Estey EH (2003). Revised recommendations of the International Working Group for Diagnosis, Standardization of Response Criteria, Treatment Outcomes, and Reporting Standards for Therapeutic Trials in Acute Myeloid Leukemia. J Clin Oncol.

[30] Tallman MS, Andersen JW, Schiffer CA, Appelbaum FR, Feusner JH, Ogden A (1997). All-trans-retinoic acid in acute promyelocytic leukemia. N Engl J Med.

[31] Sanz MA, Martin G, Gonzalez M, Leon A, Rayon C, Rivas C (2004). Risk-adapted treatment of acute promyelocytic leukemia with all-trans-retinoic acid and anthracycline monochemotherapy: a multicenter study by the PETHEMA group. Blood.

[32] Sanz MA, Grimwade D, Tallman MS, Lowenberg B, Fenaux P, Estey EH (2009). Management of acute promyelocytic leukemia: recommendations from an expert panel on behalf of the European LeukemiaNet. Blood.

[33] Russell N, Burnett A, Hills R, Betteridge S, Dennis M, Jovanovic J (2018). Attenuated arsenic trioxide plus ATRA therapy for newly diagnosed and relapsed APL: long-term follow-up of the AML17 trial. Blood.

[34] Kutny MA, Alonzo TA, Abla O, Rajpurkar M, Gerbing RB, Wang YC (2022). Assessment of Arsenic Trioxide and All-trans Retinoic Acid for the Treatment of Pediatric Acute Promyelocytic Leukemia: A Report From the Children’s Oncology Group AAML1331 Trial. JAMA Oncol.

[35] Mamez AC, Raffoux E, Chevret S, Lemiale V, Boissel N, Canet E (2016). Pre-treatment with oral hydroxyurea prior to intensive chemotherapy improves early survival of patients with high hyperleukocytosis in acute myeloid leukemia. Leuk lymphoma.

[36] Petti MC, Tafuri A, Latagliata R, Aloe Spiriti MA, Montefusco E, Mancini M (2003). High-dose hydroxyurea in the treatment of poor-risk myeloid leukemias. Ann Hematol.

[37] Montesinos P, Diaz-Mediavilla J, Deben G, Prates V, Tormo M, Rubio V (2009). Central nervous system involvement at first relapse in patients with acute promyelocytic leukemia treated with all-trans retinoic acid and anthracycline monochemotherapy without intrathecal prophylaxis. Haematologica.

[38] Wang H, Cao F, Li J, Li L, Li Y, Shi C (2014). Arsenic trioxide and mannitol for the treatment of acute promyelocytic leukemia relapse in the central nervous system. Blood.

